# Editorial

**DOI:** 10.1002/sim.10273

**Published:** 2025-01-07

**Authors:** Robert W. Platt, Nigel Stallard, Lisa McShane, Ben Van Calster, Paul S. Albert

**Affiliations:** ^1^ McGill University Montreal Canada; ^2^ University of Warwick Coventry Ireland; ^3^ National Cancer Institute Bethesda Maryland USA; ^4^ Katholieke Universiteit Leuven Universitaire Pers Leuven Leuven Belgium

It has been a while since our last editorial. We write to update you on some changes that have taken place at *Statistics in Medicine* over the last few years and to announce some upcoming ventures.

First, it is with sadness that we recognize the passing of Ralph D'Agostino Sr. last year [1]. We want to express our gratitude for his many years of dedicated service to *Statistics in Medicine*, which helped to shape the Journal into the highly regarded journal it is today. Ralph was a member of the Editorial Board at *Statistics in Medicine* from its inception in 1982; in 1990, he stepped away from the Journal for a few years and returned as Tutorials Editor in 1995. Ralph later became one of the joint Editors‐in‐Chief and remained in this role until his retirement in 2019. While Editor‐in‐Chief, Ralph continued to manage the tutorial submissions. Ralph had an extraordinary career at Boston University, holding many administrative roles including twice chairing the department of Mathematics and Statistics, and serving as director of data analysis and statistics of the Framingham Heart study from 1985 to 2015.

Second, since our last editorial there have been several changes to the Editorial team. The Editor‐in‐Chief team has changed completely since 2019. Els Goetghebeur, Simon Day, and Joel Greenhouse have all stepped down from their roles as Editor‐in‐Chief, and Lisa McShane is stepping down effective December 31, 2024, at the end of her term. We wish to thank all of them for their long service to the Journal. With the increased volume of submissions to *Statistics in Medicine*, we are pleased that the Editor‐in‐Chief group has expanded to five, and we are actively searching for a new co‐Editor‐in‐Chief. Paul Albert is responsible for Tutorials, review papers, and Special Issues, while the others (Robert Platt, Nigel Stallard, Ben Van Calster, and the new incoming editor) are focused on research papers. In addition to considering manuscripts submitted as tutorials, we have solicited manuscripts on a number of timely topics including statistical approaches for virtual autopsies, innovative uses of Mendelian randomization for causal inference, and novel designs for vaccine trials. Suzanne Thompson, our long‐standing journal administrator, retired in 2022; Suzanne's contributions to the journal are legendary, and we owe her an enormous debt of gratitude. Editorial management is now being handled fully by Wiley. The primary contact at Wiley is the Editorial Assistant, Falak Neaz, who can be reached at simeditorialoffice@wiley.com.

We have also expanded and refreshed our team of Associate Editors in recognition of the increased volume and to keep pace with changes in our field. Over the past few years, several Associate Editors have stepped down. We thank them for their dedicated and enduring service to the Journal, which helped to uphold the high standards and maintain its relevance to practicing statisticians grappling with challenging statistical issues in biomedical research. We also note with sorrow the recent passing of Associate Editor David Wheeler [2]. We welcome several new Associate Editors to the team who broaden our expertise in traditionally important topics as well as expand the coverage to additional emerging topics in biomedical statistics. We extend our appreciation to all the Associate Editors and reviewers for their hard work in maintaining the standards of the Journal. The full list of Associate Editors can be found on the journal's web page.

Our field is changing rapidly. Expanded access to data from a multitude of sources, enhanced computing power, and the ability of powerful algorithms to process data are facilitating data analyses of unprecedented type and scale. Advances in causal inference, prediction methodology, and Bayesian approaches, as well as novel methods for design and analysis of clinical trials and studies aiming to develop precision medicine tools, have had important impact on statistical practice and biomedical applications. Evaluations of performance of these new methods, by simulation or other comparative analyses, are also important to guide their use in practice, especially when applied to novel and complex data types. These emerging methods have enriched the field of statistics but do not diminish the enduring importance of sound study design, rigorous inference methods, and core statistical principles. Promoting excellent statistical practice in medical applications remains paramount in the mission of the Journal.

The January 2025 issue of Statistics in Medicine coincides with a change in our publication process. We now use Wiley's Continuous Publication (CP) model. Essentially, what this means is that articles, when typeset and finalized, are published directly into an issue. Journal issues are based on papers accepted during a pre‐specified time window and will close at the end of the publication period. This means that there will be no more Early View backlog, and that articles will appear in their final publication form more quickly following acceptance. We expect this process to lead to a more efficient publication process.

Finally, we thank all our authors and readers. The articles and our readership sustain the vibrance and relevance of the Journal.

## Conflicts of Interest

The authors declare no conflicts of interest.

## Data Availability

Data sharing is not applicable to this article as no new data were created or analyzed in this study.
